# Melatonin and the Prevention and Management of Delirium: A Scoping Study

**DOI:** 10.3389/fmed.2017.00242

**Published:** 2018-01-08

**Authors:** Sin Wei Choy, Aun Chian Yeoh, Zhao Zheng Lee, Velandai Srikanth, Chris Moran

**Affiliations:** ^1^Department of Aged Care, Alfred Health, Melbourne, VIC, Australia; ^2^Department of Geriatric Medicine, Princess Alexandra Hospital, Woolloongabba, QLD, Australia; ^3^Department of Medicine, Peninsula Health, Frankston, VIC, Australia; ^4^Peninsula Clinical School, Central Clinical School, Monash University, Frankston, VIC, Australia

**Keywords:** delirium, melatonin, melatonin agonist, ramelteon, prevention, management

## Abstract

**Background:**

The therapeutic benefit of melatonin in the prevention and treatment of delirium is uncertain.

**Objective:**

To perform a scoping study to describe the existing literature regarding the use of melatonin and ramelteon in the prevention and treatment of delirium.

**Methods:**

We performed a scoping study using the Arksey and O’Malley framework to explore our objective. Two independent panels searched MEDLINE, OVID, EMBASE, PubMed, Google Scholar, and Cochrane Library for relevant articles up to November 2017 describing the use of melatonin and ramelteon in the prevention or management of delirium. We extracted relevant summary data from the studies and attempted to draw conclusion regarding benefit.

**Results:**

We summarized evidence from 20 relevant articles. There were a total of nine articles: five randomized controlled trials (RCTs), two retrospective medical record reviews, one non-randomized observational study, and one case report describing the role of either melatonin or ramelteon in preventing delirium. There were a total of 11 studies studying the role of either melatonin or ramelteon in the management of established delirium. None of these were RCT and were predominantly case series and case reports. Four of the five trials studying the effect of melatonin analogs in preventing delirium reported a beneficial effect but study heterogeneity limited any broad recommendations. Similarly, the lack of any well-designed trials limits any recommendations regarding the effect of melatonin analogs in treating delirium.

**Conclusion:**

Large, well-designed clinical trials are required to explore the potential beneficial effects of melatonin and ramelteon on delirium prevention and management.

## Introduction

Delirium is defined as a transient change to attention and cognition that develops over a short period, is fluctuating in nature, and commonly involves disruption of the sleep–wake cycle (1, 2). It is a common condition affecting older people and is associated with significant mortality and morbidity (3). At least 10% of older patients have delirium at the time of admission to hospital and, depending upon the method of detection, between 14 and 56% experience delirium at some time during hospitalization (3).

Management of agitation and combative behavior is often a challenging aspect of delirium treatment. Although the management of these behaviors can be achieved by non-pharmacological measures, specific medications are sometimes necessary. Pharmacological interventions to manage delirium include psychotropic medications such as antipsychotics and benzodiazepines. Such medications are used commonly, despite the results of a recent systematic review not supporting their use in the treatment of delirium in older hospitalized adults (4). Benzodiazepines are also sometimes used, especially in cases of benzodiazepine and alcohol withdrawal or when antipsychotics are contraindicated (5, 6). Both of these drugs classes are associated with an increased risk of substantial harm (such as oversedation and falls) and may prolong delirium duration (7–9). This has led to the search for other pharmacological agents with a reduced side-effect profile that may improve delirium symptoms and reduce the use of potentially harmful agents.

Melatonin is an important neurotransmitter that regulates the sleep–wake cycle, facilitating sleep initiation and sleep maintenance as well as controlling the timing of sleepiness and wakefulness (10). Disruption of the sleep–wake cycle (11) and dysregulation of the circadian rhythm that controls the sleep–wake cycle may play a prominent role in the development of delirium (2, 12). Although the pathophysiology of delirium remains unclear with multiple inflammatory and cholinergic pathways likely involved, it appears that tryptophan and particularly melatonin may be important (13–21). The results from observational studies, suggest people with delirium have lower plasma (22) and salivary melatonin (23) than those without delirium. Furthermore, in one small study (*n* = 31) of people with delirium (mean age 84 years), those with hypoactive delirium (*n* = 10) had greater urinary concentration of melatonin metabolites than those with hyperactive delirium, suggesting the circulating concentration of melatonin may play a role in the delirium motor subtype (24).

Melatonin acts *via* melatonin receptors present in the suprachiasmatic nuclei (SCN) (25) and promotes sleep by attenuating the wake-promoting signal from the SCN (26, 27). Melatonin binds to three main receptors (MT1, MT2, and MT3) that appear to have important distinct but overlapping functions (17). Similarly, the choice of agent used to target melatonin receptors may be important. Ramelteon is a synthetic analog of melatonin (28, 29) and in addition to having a longer half-life than melatonin, has a sixfold higher affinity for MT1 and a threefold higher affinity MT2 receptors (30).

There is increasing interest in the role of exogenous melatonin and ramelteon in the prevention and management of people patients with delirium (28, 29, 31). A number of literature reviews have examined the role of melatonin agonists in the prevention of delirium (32, 33) and management of people with delirium (34) but have not been able to draw firm conclusions. We proposed to perform an updated search using a scoping review framework to allow us to look at the literature more broadly and attempt to describe the state of what is currently known in an attempt to guide clinical trial development.

## Methods

We used the widely accepted scoping review framework of Arksey and O’Malley to conduct and report this scoping review (35, 36). As recommended by this framework, we combined a broad research question with a defined scope of inquiry. Our broad research question was “what is known from the existing literature about the use of melatonin and ramelteon in the prevention of and management of people with delirium?” within the scope of hospital inpatients over 50 years of age. Based on our previous knowledge, we felt that prevention of delirium and management of delirium were quite distinct outcomes that were possibly the result of different pathophysiological pathways and therefore made an *a priori* decision to group studies into two distinct outcome groups: studies with delirium prevention as the primary outcome and studies with a reduction in delirium severity as the primary outcome. We purposefully kept the definition of delirium severity broad and included (but not limited to) behavior measurement and delirium resolution as potential outcome measures. In keeping with a scoping review (36), the review process was iterative to ensure a wide breadth of potentially relevant studies were identified. The purpose of this scoping review was to identify gaps in the literature to guide the design of a clinical trial examining the role of melatonin in the prevention and management of people with delirium.

### Identification of Relevant Studies

Two separate panels performed an English language search of MEDLINE, OVID, EMBASE, PubMed, Google Scholar, and Cochrane Library (1996+) in November 2017; the search terms were as follows: “delirium and melatonin”; “delirium and melatonin agonist”; “delirium and ramelteon.”

A hand search of the references of extracted articles was conducted to identify studies not captured in the electronic database searches. The reference lists of any relevant literature reviews, including Cochrane Reviews, were also hand searched to identify studies not captured other searches. As the inclusion criteria were purposefully broad, all potentially relevant studies were discussed with the senior author (Chris Moran) prior to inclusion. Reviewers met frequently to clarify study selection criteria and if necessary, refine search criteria. Once completed, the two panels shared lists of identified studies to ensure adequate capture of all suitable studies.

### Study Selection

All articles were screened by two panels and included for full text review if the abstract met the following inclusion criteria: original randomized controlled trials (RCTs), observational studies, case series or case reports study protocols, commentaries, reviews, guidelines, recommendations, database reviews, population-based reviews, systematic reviews, and meta-analysis, were written in English, and included participants aged 50 years and over.

### Data Extraction and Analysis

The selected articles were classified into two categories: (1) melatonin/ramelteon and delirium prevention and (2) melatonin/ramelteon and delirium treatment. Data extracted from each article included the following: manuscript details (primary author and publication year); study characteristics (aim, design, duration, clinical setting, country of study, and number of patients in each group); patient characteristics [mean age, population sample type (e.g., perioperative or general medical)]; intervention (description and duration of melatonin or ramelteon use); and outcome measures (and how these were defined). The data were charted as presented in the results tables.

## Results

### Searching and Screening

**Figure 1:**
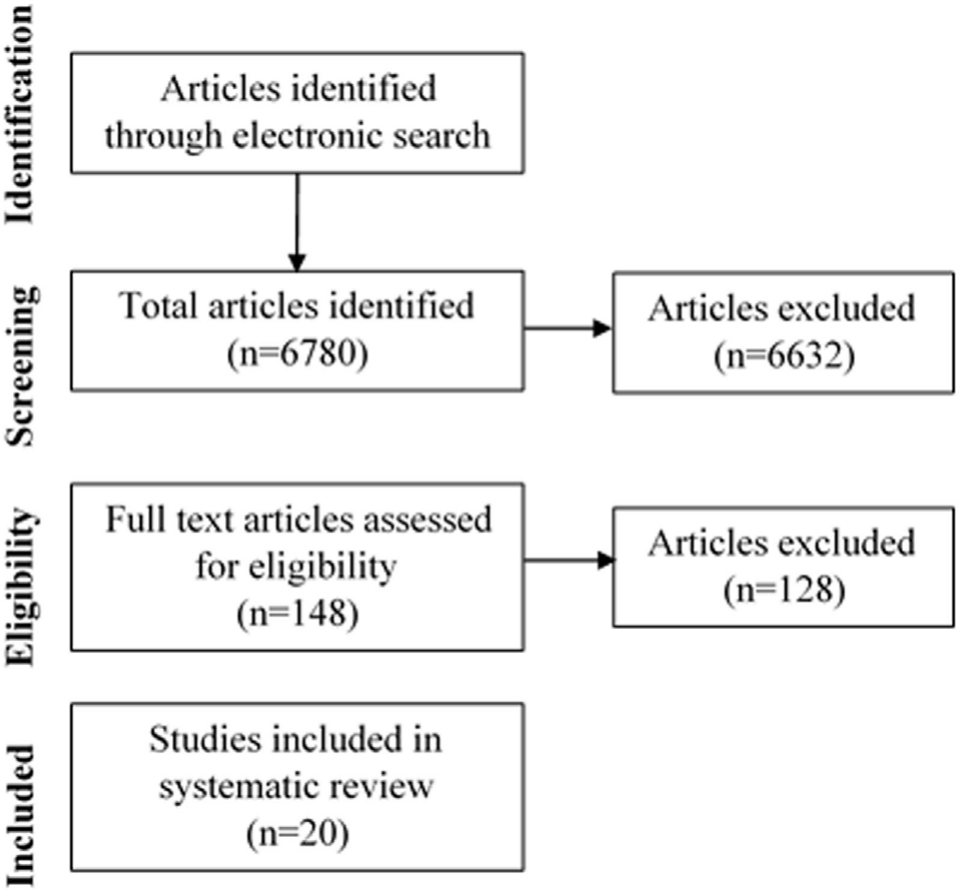
Flow diagram of selection of studies examining the association of melatonin or ramelteon on delirium.

The screening and selection process of the articles included in this review is displayed in Figure 1. The search strategy initially yielded 6,780 articles. A total of 148 articles were deemed appropriate for full text review. Following this, a further 128 studies were excluded due to a lack of relevancy and a total of 20 articles were included in this literature review.

### Study Characteristics

A total of 20 studies were identified and included in the scoping review. There were 9 studies examining the role of melatonin or ramelteon in the prevention of delirium and 11 studies examining the role in delirium management. The final review included 5 RCTs, 1 prospective observational study, 4 retrospective observational studies, and 10 other articles containing case reports or case series. Publication date of the included articles ranged from 2002 to 2017. The studies were conducted in countries including Japan, The Netherlands, Canada, Poland, China, USA, and Egypt, with Japan accounting for the majority of the studies. The studies ranged in size from a case study of one patient to large studies with 500 participants. All studies were performed in an inpatient setting. The mean age of study participants was approximately 75 years and the proportions of males and females were similar.

#### Melatonin, Ramelteon, and Delirium Prevention

There were a total of nine articles (five RCTs, two retrospective medical record reviews, one non-randomized observational study, and one case report) describing the role of either melatonin or ramelteon in preventing delirium (37–45) summarized in Table 1. Six of these examined the effect of melatonin (37–40, 42, 44) and three, ramelteon (41, 43, 45).

**Table 1:** Summary of studies using melatonin or ramelteon to prevent delirium.

Study	Study design	Sample size (*n*)	Study setting	Patient characteristics	Delirium diagnosis tool	Treatment	Results	Adverse effects (*n*)
(44); China	Double-blind RCT	Total (139) Control (70) Melatonin (69)	Elective hip arthroplasty	Mean age (years) – 74.6 in control group – 74.5 in melatonin group Gender (M/F): – 28/42 in control group – 25/44 in melatonin group Patients with psychiatric or neurological diseases (including Alzheimer’s disease) were excluded Delirium subtype not stated	Folstein MMSE	1 mg melatonin or placebo 1 hour before bedtime a day before surgery and for another 5 consecutive days post surgery	Delirium not formally diagnosed. Outcome: “Post Operative Cognitive Decline” – Melatonin was associated with unchanged MMSE score post surgery, whereas MMSE score decreased significantly at days 1, 3, and 5 after surgery (*F* = 3.595, *p* < 0.05) in the control group.	Similar side effects in both groups. Melatonin Dizziness (11) Headache (8) Paresthesia (9) Nausea (14) *p* = 0.994

(40); The Netherlands	Multi-center, double-blind RCT	Total (378) Placebo (192) Melatonin (186)	Acute surgery for hip fracture	Mean age: 84 years Gender (M/F) – 62/130 in placebo group – 53/133 in melatonin group Background of MCI – 106/192 in placebo group – 104/186 in melatonin group Delirium subtype not stated	DSM-IV; DOSS	3 mg melatonin or placebo for 5 consecutive evenings	– Melatonin treatment did not reduce incidence of delirium – Similar delirium severity, LOS, mortality, and cognitive or functional outcomes at 3 months – A smaller proportion of patients had longer episode of delirium (>2 days) in the melatonin group (25.5 vs. 46.9%, *p* = 0.02).	Not reported

(41); Japan	Multi-center, blind RCT	Total (67) Placebo (34) Ramelteon (33)	Intensive care units and acute wards	Mean age: 78 years Gender (M/F): – 11/23 in placebo group – 16/17 in ramelteon group Delirium subtype not stated	DSM-IV	8 mg ramelteon or placebo every night for 7 days	– Ramelteon was associated with lower incidence of delirium (3.0 vs. 32.0%; *p* = 0.003) – Rate of delirium was also lower in patients without a history of delirium (0 vs. 30%; *p* = 0.001) – There was no difference in delirium severity, changes in APACHE II score^b^ and sleep parameters	Not specifically reported Described as: “well tolerated”

(39); Canada	Double-blind RCT	Total (122) Placebo (61) Melatonin (61)	Acute medical unit	Mean age: 84.5 years Gender (M/F) – 24/37 in placebo group – 28/33 in melatonin group Background of MCI or dementia: – 20/61 in placebo group – 23/61 in melatonin group Delirium subtype not stated	CAM	0.5 mg melatonin or placebo every night for 14 days or until discharge	– Melatonin was associated with a lower risk of delirium (12.0 vs. 31.0%, *p* = 0.014) – Melatonin did not decrease delirium severity, LOS, restraint, sedative use or mortality	Nightmare (1) Hallucinations (1)

(38); Egypt	Double-blind RCT	Total (203) Control (49) Melatonin (53) Midazolam (50) Clonidine (51)	Scheduled hip arthroplasty under spinal anesthesia; compared with 3 groups: control, midazolam, clonidine	Mean age (years) – 72.3 in control group – 70.4 in melatonin group – 69.9 in midazolam group – 71.5 in clonidine group Gender (M/F) – 22/27 in control group – 24/29 in melatonin group – 26/24 in midazolam group – 27/24 in clonidine group Dementia excluded Delirium subtype not stated	AMT	5 mg melatonin at sleep time and 90 min before operative time (compared to no premedication; 7.5 mg midazolam and 100 µg clonidine given at similar times); patients with postoperative delirium received 5 mg melatonin for 3 days	– Melatonin was associated with a lower rate of postoperative delirium (9.43%; *p* = 0.003) compared to 33% in control group; 44% in midazolam group and 37% in clonidine group – Melatonin treated 58.06% of patients with postoperative delirium (36/62) with no significant group differences	Not reported

(42); Poland	Single-center prospective non-randomized observation study	Total (500) Control (250) melatonin (250)	Various elective and urgent cardiac surgery	Mean age (years) – 65.2 in control group – 64.3 in melatonin group Gender (M/F): – 171/79 in control group – 179/71 in melatonin group All had hyperactive delirium	CAM-ICU; RASS	5 mg melatonin vs. control, given the evening before surgery and continued until day 3 postoperative	– Melatonin was associated with lower incidence of postoperative delirium (8.4 vs. 20.8%, *p* = 0.001) – Predictors of delirium in melatonin group were age (*p* = 0.001) and higher EuroSCORE II^a^ value (*p* = 0.001)	Not reported

(43); Japan	Retrospective analysis of medical records	Total (65) minor transquilizers (24) ramelteon (25) ramelteon and suvorexant (16)	Post pharygo-laryngectomy with esophagectomy	Mean age: 66 years Gender (M/F): 56/9 Delirium subtype not stated	DSM-V	Minor transquilizers (mainly zolpidem) vs. 8 mg ramelteon vs. ramelteon and 20 mg suvorexant throughout the duration of hospital stay	– Ramelteon with or without suvorexant was associated with a decreased rate of postoperative delirium compared to minor transquilizers (*p* = 0.001) – 8/24 patients (33.3%) on minor transquilizers had delirium in comparison to 1/25 (4.0%) on ramelteon and 0/16 on ramelteon and suvorexant (0.0%) – Most delirium cases occurred between days 1 and 3 postoperatively	Not reported

(45); Japan	Retrospective analysis of medical records	Total (82) Control (58) Ramelteon (24)	Post pulmonary resection of lung cancer (ICU and inpatient ward setting)	Mean age (years) – 76.5 in control group – 79 in ramelteon group Gender (M/F): – 43/15 in control group – 21/3 in ramelteon group Delirium subtype not stated	ICDSC	8 mg ramelteon was administered daily for 7 days after surgery vs. control	– Ramelteon was associated with a trend of lower incidence of ICDSC^c^ events but was not statistically significant (recovery index: 0.25 + 0.74 vs. 1.58 + 4.93, *P* = 0.061) – Time to complete recovery of delirium was 1 day and 8 days in the ramelteon and control groups, respectively	One patient in ramelteon group had dizziness on day 2

(37); Japan	Case report	1 case	Open debridement of an infected knee joint	78-year-old man with a past history of postoperative delirium Delirium subtype not applicable	None	2 mg melatonin slow release for 3 nights postoperatively	Delirium did not develop during 5-day hospitalization	Not reported

*RCT, randomized controlled trial; MCI, mild cognitive impairment; M/F, male/female; LOS, length of stay; AMT, Abbreviated Mental Test; CAM, Confusion Assessment Method; CAM-ICU, Confusion Assessment Method for the ICU; DSM-IV, Diagnostic and Statistical Manual of Mental Disorders-Fourth Edition; DSM-V, Diagnostic and Statistical Manual of Mental Disorders, Fifth Edition; DOSS, The Delirium Observation Screening Scale; Folstein MMSE, Folstein Mini-Mental State Examination*.

*^a^European System for Cardiac Operative Risk Evaluation: risk model designed to calculate the risk of death after a cardiac operation*.

*^b^Acute Physiology And Chronic Health Evaluation II: ICU scoring system designed to measure severity of disease in patients admitted to ICU*.

*^c^Intensive Care Delirium Screening Checklist: 8-item delirium screening tool used in ICU*.

##### Melatonin and Delirium Prevention

Four RCTs have examined the efficacy of melatonin in preventing delirium with contradictory results (38, 39, 40, 44).

The earliest study was a double-blind RCT of 203 people ≥65 years of age scheduled for hip arthroplasty under spinal anesthesia investigating the efficacy of three different preoperative sedative medications including melatonin to prevent the incidence of postoperative delirium (38). Patients were allocated into four different treatment groups: Group 1 (control) received no sedation; Group 2 received 5 mg melatonin; Group 3 received 7.5 mg midazolam; and Group 4 received 100 µg clonidine. Medications were administered orally at sleep time on the night of surgery as well as 90 min before surgery. The Abbreviated Mental Test (AMT) was performed on the day of operation and for three consecutive postoperative days. Delirium was defined as a new AMT score <8. Of the 203 patients who completed the study (mean age ~72 years, 51% female), the rate of postoperative delirium was lower in the melatonin group (9%, *n* = 5/53) than the other groups [control group: 33%, *n* = 16/49; midazolam group: 44%, *n* = 22/50; clonidine group: 37%, *n* = 19/51 (*p* = 0.003)] (38).

In a second double-blind RCT, 122 acute medical unit inpatients (mean age ~85 years, 57% female) were randomized to receive either 0.5 mg melatonin or placebo every night for 14 days or until discharge (39). In this study, delirium was defined according to the Confusion Assessment Method criteria (46). Those given melatonin had a lower incidence of delirium (12 vs. 31%, *p* = 0.01). When adjusted for the presence of other co-morbidities such as dementia the beneficial effect of melatonin remained (odds ratio = 0.19, 95% CI: 0.06–0.62). Rates of delirium severity, length of stay, need for sedation or restraints, and mortality were similar between the two groups (39).

In a larger double-blind RCT of preoperative patients with hip fracture, 378 patients (mean age ~84 years, 70% female) were given either 3 mg of melatonin or placebo for five consecutive days starting within 24 h after admission (40). Delirium was diagnosed using the *DSM*, fourth edition (DSM-IV) criteria (1). The incidence of delirium, duration and severity of delirium, and number of sedative/psychotropic medications was similar between the two groups. After 3 months of follow-up, cognitive function, functional status, and mortality rates were also similar between the two groups (40).

A recent double-blind trial randomized 139 patients (mean age ~75 years, 62% female) awaiting elective hip arthroplasty to 1 mg of melatonin or placebo an hour before bedtime on the day before surgery and for another 5 days postoperatively (44). The primary outcome was not a formal diagnosis of delirium but “early postoperative cognitive decline” measured as change in Folstein Mini-Mental State Examination (MMSE) scores. When compared to the melatonin arm, patients given placebo had lower MMSE scores at days 1, 3, and 5 (*p* < 0.05), worse subjective sleep quality, general well-being, and fatigue (44).

In a Polish study (mean age ~65 years, 30% female), a group of 250 people receiving standard care were compared to subsequent group of 250 people given 5 mg of melatonin the evening before elective or urgent cardiac surgery of various types and for 3 nights postoperatively (42). In this observational, non-randomized study, the incidence of postoperative delirium [measured using the Confusion Assessment Method for the Intensive Care Unit (CAM-ICU)] was lower in the melatonin group (8.4%) than the control group (20.8%) (*p* = 0.001) (42).

We also identified a single case report describing the absence of the development of delirium in a 78-year-old man with a history of postoperative delirium who underwent an open debridement of an infected knee joint. After receiving three nights of treatment with 2 mg of melatonin, the authors reported he remained alert and oriented after surgery (37).

##### Ramelteon and Delirium Prevention

There were three studies examining the effect of ramelteon on delirium prevention (41, 43, 45). One study (41) reported a multi-center single-blind RCT that randomized 67 patients (mean age 78 years, ~60% female) with “serious medical problems” admitted to the intensive care unit or regular acute wards to receive either 8 mg of ramelteon or placebo every night for 7 days. In this study, those who received ramelteon had a lower incidence of delirium [defined according to *DSM-IV* criteria (1)] than those who received placebo (3.0 vs. 32.0%, *p* = 0.003) (41).

Two studies described the results of retrospective analyses of medical records. The largest of these studies (*n* = 82, mean age ~77 years, 22% female) examined the occurrence of delirium using the validated Intensive Care Delirium Screening Checklist in a sample of people admitted to ICU or inpatient wards following pulmonary resection of lung cancer (45). The authors reported a trend for a lower incidence of delirium and a lesser intensity of delirium symptoms in the group that received ramelteon (*n* = 24) than those in the control group (*n* = 58) but these differences were not statistically significant (45). Another retrospective analysis of medical records performed in postoperative patients compared the incidence of delirium defined according to DSM-V criteria in three separate arms: 8 mg ramelteon, tranquilizers, and a combination of 8 mg ramelteon and 20 mg suvorexant (43). When compared to those taking tranquilizers (*n* = 24), those taking ramelteon (*n* = 25) or both ramelteon and suvorexant (*n* = 16) had lower incidences of delirium (33, 4, and 0%, respectively) (43).

#### Melatonin, Ramelteon, and Treatment of Established Delirium

There were a total of 11 studies studying the role of either melatonin or ramelteon in the management of established delirium (37, 47–56). Table 2 summarizes the published literature examining the effect of melatonin or ramelteon on the treatment of established delirium. To date, there have been no RCT studying the role of melatonin in treating established delirium. One case report described the effect of melatonin on treatment of delirium (37). There were two retrospective medical record reviews examining the association between ramelteon use and delirium management (53, 56). There were 8 published articles, describing a total of 29 cases reporting the effect of ramelteon on the treatment of delirium (47–52, 54, 55).

**Table 2:** Characteristics of case reports of melatonin or ramelteon to treat delirium.

Study	Study design	Sample size	Study setting	Patient characteristic	Delirium measurement tool	Treatment	Results	Adverse effects
(56); USA	Single-center, retrospective cohort study of medical charts	Total (125) Control (65) Ramelteon (60)	Inpatients throughout the hospital followed up by psychiatric service	Mean age (years) – 83.7 in control group – 86 in ramelteon group Gender (M/F) – 34/65 in control group – 31/60 in ramelteon group Hyperactive delirium	DSM-V	Dose of ramelteon was not specified	– Ramelteon was associated with lower incidence of as-needed antipsychotics (60 vs. 80%, *p* = 0.001) – Ramelteon was associated with lower total doses of antipsychotics (40 vs. 14%, *p* = 0.004) – There was no between-group difference for type of antipsychotics, the distribution of total doses in milligrams administered, and total length of stay	Not reported

(53); Japan	Single-center retrospective study of medical records	Total (32) Non-ramelteon group (19) Ramelteon group (13)	Inpatients followed up by consultation-liaison psychiatry service	Mean age (years) – 79.7 in non-ramelteon group – 78.1 in ramelteon group Gender (M/F) – 7/12 in non-ramelteon group – 7/6 in ramelteon group Delirium subtype not stated	DSM-IV-TR	Adjunctive 8 mg ramelteon with antipsychotics compared with antipsychotics monotherapy	– Ramelteon was associated with less duration of delirium of delirium (6.6 vs. 9.9 days, *p* = 0.048) – Ramelteon was also associated with smaller total amount of antipsychotics dosage (444.5 vs. 833.4 mg, *p* = 0.044)	Not reported

(50); Japan	Retrospective case review	7 cases	All with acute stroke (5 with cerebral infarct; 2 with hemorrhage)	Mean age 76 years, 3 female, 4 male All patients had hyperactive delirium	CAM-ICU; RASS	8 mg ramelteon	– Improvement of delirium including improvement of RASS score in 4 patients – All patients had better sleep	Not reported. Ramelteon was “well tolerated”

(52); Japan	Case series	10 cases	8 patients with acute medical conditions; 2 with postoperative delirium	Mean age of 89.5, 3 female, 7 male All patients had hyperactive delirium	DSM; DRS-R-98	8 mg ramelteon	– Improvement of delirium based on DRS-R-98	Not reported. Ramelteon was “well tolerated”

(48); Japan	Case series	5 cases	Medical conditions (“circardian rhythm disturbance of Alzheimer’s dementia,” unclear etiology, steroid psychosis, and benzodiazepine withdrawal)	Aged 71–91 years old, 2 female, 3 male All patients had hyperactive delirium	DSM-IV-TR; DRS	8 mg ramelteon	– Marked improvement of delirium based on DRS within a day of administration – Ramelteon also improved sleep–wake cycle, attention and cognitive functions	Not reported. Ramelteon was “well tolerated”

(47); Japan	Case series	3 cases	Acute medical conditions (aspiration pneumonia, cervical spinal cord injury, pyelonephritis)	Aged 59–83 years old, all female Delirium subtype not stated; likely mixed	MDAS	8 mg ramelteon	– MDAS scores improved significantly from baseline to day 7 with ramelteon treatment	Not reported. Ramelteon was “well tolerated”

(51); Japan	Case report	1 case	Severe pneumonia; in intensive care unit	100-year-old man	DRS-R-98	8 mg ramelteon	– Improvement of delirium based on DRS-R-98	Not reported. Ramelteon was “well tolerated”
				No history of dementia				
				Hyperactive delirium				

(49); Japan	Case report	1 case	Emergency craniotomy for right temporal lobe hemorrhage, and subsequent clipping of an unruptured aneurysm	68-year-old lady Hyperactive delirium not responding to 150 mg thiapride	MDAS	8 mg ramelteon (changed from triazolam) and 7.5 g Yi-gan san	– Improvement of delirium based on MDAS score	Not reported

(55); Japan	Case report	1 case	Pneumonia	75-year-old man Hyperactive delirium	DSM-IV-TR; DRS-R-98	4 mg ramelteon titrated to 8 mg (in addition to antipsychotics)	– Improvement of delirium based on DRS-R-98	Not reported

(54); Japan	Case report	1 case	Stage IV cancer of pharynx	63-year-old man Hypoactive delirium (multifactorial causes) 30 days after chemotherapy and radiotherapy	DSM-V; DRS	4 mg ramelteon	– Improvement of delirium based on DRS including better sleep–wake cycle at day 2 post ramelteon administration	Not reported. Ramelteon was “well tolerated”

(37); Japan	Case report	1 case	Hip pinning procedure for hip fracture	53-year-old man who developed delirium 2 days postoperatively requiring restraints while on PCA, not responding to antipsychotic or benzodiazepine	None	2 mg melatonin slow release was given day 4 post surgery, and continued for 3 more nights	– Patient had better sleep the same night melatonin was given and was oriented the next day	Not reported
				Delirium subtype not stated; likely mixed				

*MDAS, Memorial Delirium Assessment Scale; RASS, Richmond Agitation and Sedation Scale; CAM-ICU, Confusion Assessment Method for the ICU; DRS, Delirium Rating Scale; DRS-R-98, Delirium Rating Scale-revised-98; DSM, Diagnostic and Statistical Manual of Mental Disorders; DSM-IV-TR, Diagnostic and Statistical Manual of Mental Disorders-Fourth Edition (Text Revision); DSM-V, Diagnostic and Statistical Manual of Mental Disorders, Fifth Edition*.

##### Melatonin and Delirium Treatment

In one case report of a 53-year-old postoperative male with severe delirium requiring antipsychotics, benzodiazepines, and physical restraints, the authors reported that the administration of 2 mg of slow release melatonin resulted in better sleep the night he was administered melatonin and that he was oriented the next day (37).

##### Ramelteon and Delirium Treatment

Of the 10 published articles reporting the use of ramelteon on established delirium (47–56), there were two retrospective observational studies (53, 56), four case series (47, 48, 50, 52) and four reports describing a single case (49, 51, 54, 55).

Two studies were single-center retrospective cohort studies based on medical record review. In the largest study (*n* = 125), patients followed by psychiatry services (mean age ~85 years, 52% female) with hyperactive delirium (based on DSM-V criteria) underwent chart review (56). Those patients given ramelteon had lower use of as-needed antipsychotics (60 vs. 80%, *p* = 0.001) than those who did not receive ramelteon (56). A smaller study (*n* = 32), also based on a retrospective medical chart review of patients followed up by a consultation-liaison psychiatry service compared those with delirium (according to DSM-IV-TR criteria) taking antipsychotics to a group with delirium taking both antipsychotics and ramelteon (53). The authors reported that the addition of ramelteon was associated with less duration of delirium (6.6 vs. 9.9 days, *p* = 0.048) and a smaller total amount of antipsychotics dosage (444.5 vs. 833.4 mg, *p* = 0.044) (53).

In a case series reporting the use of ramelteon in three female patients (aged 59, 66, and 83 years) with delirium presenting with different acute medical conditions (aspiration pneumonia, cervical spinal cord injury, and pyelonephritis), the authors reported that the administration of 8 mg/day ramelteon was associated with improvement in delirium severity as measured using the Memorial Delirium Assessment Scale score (MDAS) (47, 57).

One case series described five cases of delirium in people (2 females, 3 males) aged between 71 and 91 years old being successfully treated within 1 day of the administration of 8 mg of ramelteon (48). The authors described large improvements in the Delirium Rating Scale after a single dose of ramelteon and reported the delirium to have resolved (48).

In another case series, the authors described a retrospective review of seven older people (mean age 76 years, 3 females, 4 males) given 8 mg of ramelteon with delirium and insomnia after acute stroke (50). The study reported that all those who received ramelteon showed improvement in either the Richmond Agitation and Sedation Scale or sleep quality within 1 week of administration (50).

A further case series described 10 patients (mean age 90 years, 3 females, 7 males) with delirium (8 people with acute medical conditions, 2 post surgery) who were administered 8 mg of ramelteon (52). The authors reported improvement of delirium based on Delirium Rating Scale-revised-98 (DRS-R-98) at day 3 of treatment in seven out of the 10 patients, with the remaining three not responding to treatment (52).

One single case report described a 100-year-old man with delirium who did not tolerate low dose risperidone and was over-sedated (51). On the ninth day of his admission, he was commenced on 8 mg ramelteon daily and the authors reported that the patient’s delirium severity score (DRS-R-98) was improved at day 13 of his admission (51).

Another single case report described a 68-year-old lady with postoperative delirium after neurosurgery who did not respond to thiapride. She was commenced on an 8 mg ramelteon and thiapride was changed to 7.5 g Yi-gan san daily. The authors reported delirium improvement based on MDAS score at 7 days (49).

One case report described a 75-year-old man with Alzheimer’s disease, admitted to hospital with pneumonia complicated by an episode of delirium which did not respond to antipsychotic medicine with fluctuating severe psychomotor agitation and oversedation (55). The authors reported that the administration of 4 mg ramelteon at day 10 resulted in improvement of his sleep–wake cycle on the same day. The ramelteon dose was increased to 8 mg at day 18 and his delirium severity score (measured using the DRS-R-98) improved and his delirium resolved at day 21 (55).

A further single case report described a 63-year-old man with pharyngeal cancer who developed a hypoactive delirium as per the *DSM-V* criteria (54). The delirium did not respond to non-pharmacological interventions and after 9 days, 4 mg ramelteon was commenced with good clinical effect. The authors also reported improvement in the patient’s sleep–wake cycle 2 days later (54).

## Discussion

### Prevention of Delirium

There is a lack of high quality data supporting the role of melatonin and ramelteon in the prevention of delirium. To date, there have been only four published RCT (38, 39, 40, 44) examining the role of melatonin and one examining the role of ramelteon (41) in the prevention of delirium. Although four of the five trials studying the effect of melatonin analogs in preventing delirium reported a beneficial effect (38, 39, 41, 44), the heterogeneity of the study designs make broad recommendations impossible at this stage. There were variations in the study sample setting, e.g., acute medical (39), acute hip fracture (40), post elective hip arthroplasty (38, 44) as well as the tools used to diagnosis delirium, with some using diagnostic tools not validated for this purpose, e.g., AMT (38) and MMSE (44).

The other studies included a non-randomized study (42), two retrospective analyses of medical records (43, 45), and a single case report (37). All of these studies have a high risk of bias inherent in their study design. The lack of randomization prevents adequate control of potential confounders and introduces a potential selection bias into the choice of which patients receive melatonin. Retrospective chart reviews are limited by the quality and accuracy of documentation. This is particularly of concern given that delirium is frequently under-recognized and reported (58). Similarly a report describing a single case of a person who did not develop delirium following melatonin administration is limited in its ability to attribute causality.

In all the studies, there were wide variations in the dose (0.5–5 mg) and duration of melatonin use (5–14 days). Given the suggestion that melatonin plays a role in the motor subtype of delirium (24), it may be relevant to include the motor subtype in the reporting of results. However, in general, delirium motor subtype was poorly reported in all the published studies with only one of the nine published studies describing this potentially important detail (42).

Of the nine published studies, eight reported results supportive of a beneficial effect of melatonin analogs in preventing the incidence of delirium (37, 38, 39, 41, 42, 43, 44, 45). These results encourage further research into this area but highlight the need for well-designed trials to improve confidence in these early results. Important questions regarding the choice of agent (melatonin or ramelteon), drug dose, and duration and the sample of people who may benefit from remain unanswered. Future RCTs will need to include well-validated tools to identify delirium and ensure they are adequately powered to identify between-group differences. A number of published study protocols (59, 60), including feasibility studies (61, 62), suggest that such improvements in the available evidence of the efficacy of melatonin analogs to prevent delirium are forthcoming.

### Management of Delirium

The evidence supporting the role of melatonin or ramelteon in the management of people with established delirium is of poor quality with a lack of any well-designed, RCT. Two studies were based on a single-center retrospective review of medical records (53, 56) and the remainder were case reports (37, 49, 51, 54, 55) and case series (47, 48, 50, 52). All of these study designs are limited by a lack of clinician blinding to the intervention and the use of different measures of delirium improvement. This prevents the drawing of any firm recommendations for clinical practice. The measurement of delirium improvement ranged from clinician impression of an improvement in sleep (37), through validated delirium severity scoring tools, to examining the doses of antipsychotics used to manage behavior (56). Most studies reported patients to have a hyperactive motor subtype of delirium while some did not report subtype at all (37). As previously discussed, motor subtype may be important, as the effectiveness of melatonin analogs may be dependent upon motor subtype. Case reports and cases series study designs are unable to attribute any improvements directly to the administration of melatonin or ramelteon due to the lack of a control arm with any improvements seen possibly to due to the resolution of delirium due to other delirium management measures.

All of the published studies reported a positive effect of melatonin or ramelteon in the management of people with established delirium. Although the study design and the potential for reporting bias and publication bias may play a role, the results are generally encouraging of the development of further research into this area. This scoping study highlights the need for well-designed RCT to explore this further. Similar to the issues with the role of melatonin agonists in preventing delirium, questions regarding agent choice, dose, duration, and outcome measures still remain. Published trial protocols suggest that this is an ongoing area of research (63) and efforts to develop agreed outcome measures for trials of interventions to prevent or treat delirium are being developed (64).

This scoping review has some limitations. We have collated the evidence on the topic of melatonin and ramelteon in the prevention and management of delirium but have not performed in-depth critical appraisal of all of the included articles in keeping with the scoping review framework. We excluded article preceding 1996 and therefore may have under-reported the extent of the available published literature.

## Conclusion

We have identified and collated the available evidence describing the use of melatonin and ramelteon to prevent or manage people with delirium. We conclude that there is a paucity of well-designed trials to address either outcome but the available evidence encourages further research. Large well-designed RCT are required to better understand the potential of melatonin or ramelteon to manage a common and serious syndrome.

## Key Points

- Delirium is a common clinical syndrome with significant mortality and morbidity.
- Melatonin is an important hormone in the regulation of sleep–wake cycle and may play a role in preventing or treating delirium.
- The role of melatonin in the prevention or management of delirium is unclear, and is limited by the small number of and large variability in the design of published studies.
- Large, well-designed clinical trials exploring the potential beneficial effects of exogenous melatonin on delirium are urgently needed.

## Author Contributions

SC developed the study design, performed the literature search, screened the articles, and extracted the data. She also summarized the results and wrote the manuscript. AY and ZL assisted with the literature review and data collection. VS and CM assisted with drafting and review of the manuscript. CM also helped designed the study, revised the manuscript, and approved the final version.

## Conflict of Interest Statement

The authors declare that the research was conducted in the absence of any commercial or financial relationships that could be constructed as a potential conflict of interest.
